# Brainstem Tuberculous Abscess in a 63‐Year‐Old Adult With Unrepaired Tetralogy of Fallot

**DOI:** 10.1155/carm/8475963

**Published:** 2026-06-02

**Authors:** Jessica S. Yang, Flora Y. Kurtz, Hans H. Liu, Benjamin Ehrlich, Gbambele Kone

**Affiliations:** ^1^ Family Medicine Wynnewood, Bryn Mawr Hospital, Bryn Mawr, Pennsylvania, 19010, USA, mainlinehealth.org; ^2^ Department of Medicine/Hospital Medicine, Bryn Mawr Hospital, Bryn Mawr, Pennsylvania, 19010, USA, mainlinehealth.org; ^3^ Department of Medicine/Infectious Diseases, Bryn Mawr Hospital, Bryn Mawr, Pennsylvania, 19010, USA, mainlinehealth.org; ^4^ Department of Neurology, Bryn Mawr Hospital, Bryn Mawr, Pennsylvania, 19010, USA, mainlinehealth.org

**Keywords:** case report, central nervous system tuberculous abscess, endocarditis, septic embolism, tetralogy of Fallot

## Abstract

**Background:**

Tetralogy of Fallot (TOF) is a congenital heart anomaly usually corrected surgically in childhood. Reports of adults with unrepaired TOF indicate that they are at high risk of endocardial infections and sequelae such as distant abscesses.

**Case Presentation:**

A 63‐year‐old man born and raised in Vietnam presented with unrepaired TOF and 5 days of dizziness, dysarthria, and left‐sided weakness. Magnetic resonance imaging (MRI) of the brain demonstrated a 2.9‐cm rim‐enhancing pontine lesion with associated edema and a punctate acute infarct in the right cerebellum. Echocardiogram of the heart revealed right‐to‐left shunting, while chest computerized tomography (CT) showed a left apical cavitary mass. Initial studies were negative for bacteremia and endocarditis. Due to clinical suspicion and a positive QuantiFERON Gold tuberculosis (TB) test, antituberculous therapy was initiated. Biopsy of the central nervous system (CNS) lesion was deferred due to risk, and therapy was continued with antituberculous agents and steroids. The patient improved with a final diagnosis of tuberculous brainstem abscess.

**Conclusion:**

This case highlights early recognition and awareness of potential complications associated with untreated TOF as well as suspicion for TB in the proper clinical setting. Complex cases involving rare diagnoses should be approached systematically, evaluating possible diagnoses in order of urgency and basing invasive procedures on patient risk–benefit.

## 1. Introduction

Tetralogy of Fallot (TOF) is a congenital heart anomaly characterized by an overriding aortic root, obstruction in the right ventricular outflow tract, right ventricular hypertrophy, and a typically large ventricular septal defect (VSD) [1]. Most individuals with TOF undergo surgical intervention in infancy, as only 3% of cases of untreated TOF survive into their 40’s [2]. Adults with unrepaired TOF are at risk for multiple complications, including pulmonary hypertension, heart failure, arrhythmias, and embolic events [3]. Emboli are most commonly systemic, such as strokes, but pulmonary emboli are also possible. Infective endocarditis is also a common complication of TOF [4] and may lead to septic embolization with abscess development in the affected sites. Due to this, as well as other predispositions, up to 11% of patients with congenital heart disease will develop brain abscesses [5], with the highest percentage in TOF patients [6]. These facts must be considered when any adult with uncorrected TOF is seen with neurologic decompensation. Our patient is one of only a few individuals diagnosed with a tuberculous brain abscess in the setting of TOF. This case is further distinguished by the patient’s advanced age, the brainstem location of the abscess, the development of a management plan without invasive diagnostic procedures, and his favorable response to empiric therapy.

## 2. Case Presentation

**FIGURE 1:**
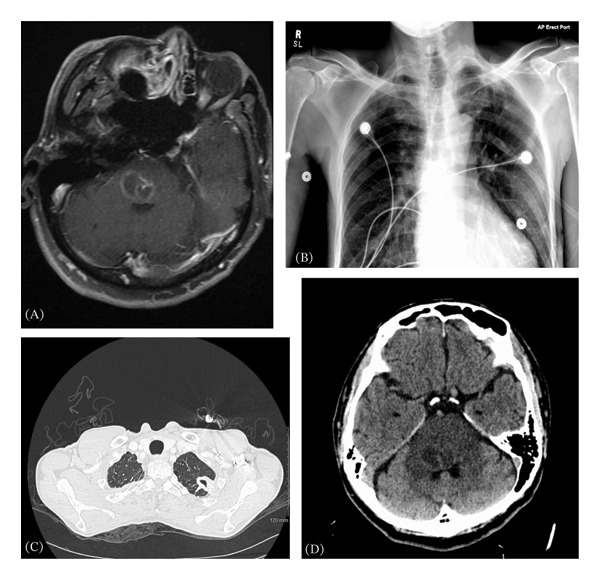
(A) Initial MRI T1 postcontrast at hospital presentation with a 2.9 × 2.2 × 1.9 cm rim‐enhancing multiloculated lesion at the pons with surrounding vasogenic edema. (B) Initial chest X‐ray on hospital admission with scattered calcified granulomas and a left lung cavitary lesion. (C) CT of chest showing a left apical cavitary lesion measuring 2.9 × 1.7 cm. (D) CT of head showing effacement of the fourth ventricle on Day 6 of hospitalization.

A 63‐year‐old male originally from Vietnam presented to a hospital in the mid‐Atlantic coastal United States with five days of progressively worsening dizziness, lightheadedness, left arm and leg weakness with numbness, dysarthria, and word‐finding difficulty. It was noted that the patient only intermittently utilized healthcare opportunities and had TOF initially diagnosed 12 years prior to presentation, at which time he had declined any treatment. He was afebrile on admission with a pulse of 60/min and normal blood pressure. He briefly desaturated to 88% on room air and was placed on 2 L/min nasal oxygen with improvement in saturation. Neurologic examination revealed right facial weakness in a lower motor neuron distribution, right ophthalmoplegia, right internuclear ophthalmoplegia (INO), left hemibody paresis, ataxia, and sensory loss with an impaired gait. Computerized tomography (CT) of the head showed a lesion in the right pons/midbrain. Magnetic resonance imaging (MRI) of the brain revealed a 2.9‐cm rim‐enhancing multiloculated lesion at the pons with surrounding vasogenic edema (Figure 1A), as well as a smaller acute right cerebellar infarct. CT angiography of the head/neck was unremarkable. Chest X‐ray showed granulomas (Figure 1B), and chest CT showed a 3.2‐cm cavitary left apical mass with extensive mediastinal collateralization (Figure 1C).

He was empirically started on vancomycin, ceftriaxone, and ampicillin for a possible CNS bacterial infection. Cerebrospinal fluid (CSF) profile showed red blood cells 10/μL, white blood cells 214/μL (4% neutrophils, 57% lymphocytes, and 39% monocytes and macrocytes), glucose 53 mg/dL (serum 97 mg/dL), and protein 76 mg/dL. CSF cytology, cultures, gram stain, acid‐fast stain, meningitis/encephalitis PCR panel, and oligoclonal bands were negative. QuantiFERON gold assay was positive, with tuberculosis (TB) antigen 3.93 for TB1 antigen‐nil (primarily CD4 T cell response) and 3.37 for TB2 antigen (CD4 and CD8 T cell response). A transthoracic echocardiogram (TTE) showed a perimembranous VSD measuring 1.1 cm with active shunting, consistent with an unrepaired TOF. On day six of hospitalization, the patient developed worsening left‐sided weakness. CT of the chest showed a left‐sided 2.9 × 1.7 cm cavity. Repeat CT of the head revealed hemorrhage of the pontine lesion and effacement of the 4th ventricle (Figure 1D). The patient was transferred to a tertiary care center’s neurointensive care unit for monitoring and stabilization, where he was intubated on arrival.

At the tertiary care center, he was started on linezolid, levofloxacin, and metronidazole as well as an antituberculous regimen with rifampin, isoniazid, pyrazinamide, and ethambutol (RIPE). He also received dexamethasone. He was evaluated by neurosurgery, and a biopsy of his intracranial lesion was deferred, given the location and risk. A bedside bronchoscopy of the left upper lobe lesion yielded bronchoalveolar lavage (BAL) with negative fungal cultures and Ziehl–Nielsen acid‐fast staining, with the left upper lobe lesion favored to represent a fibrotic cavity. All blood cultures were negative, so vancomycin, ceftriaxone, and ampicillin were discontinued. The final presumed diagnosis was CNS TB in the setting of untreated TOF.

Due to persistent dysphagia, he had a percutaneous endoscopic gastrostomy (PEG) tube placed. After a month at the tertiary care center, he was transferred back to the original hospital for continuity of care with instructions to continue RIPE, linezolid, and levofloxacin. Linezolid, pyrazinamide and ethambutol were discontinued after 9 weeks of therapy. He was continued on treatment with rifampin, isoniazid, levofloxacin, and vitamin B6, with levofloxacin continued to provide coverage for possible multidrug‐resistant TB.

**FIGURE 2:**
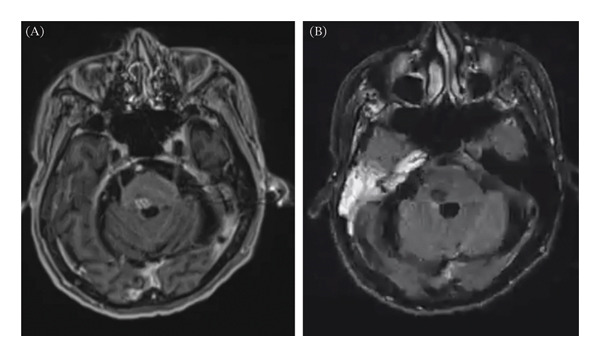
MRI brain at 3 months after initiation of treatment with TB therapy. (A) T1 postcontrast imaging with decreased size of enhancing pontine lesion now measuring < 1 cm. (B) FLAIR imaging demonstrating resolution of vasogenic edema associated with tuberculous lesion.

He was discharged to a skilled nursing facility and ultimately returned home. MRI studies of the brain after 3 months on antituberculous therapy showed improvement in the size of the abscess and surrounding edema (Figure 2A, B). At the outpatient follow‐up 6 months later, our patient had clinically improved. He had intact orientation but delayed recall. Right eye movement remained impaired, but lateral gaze was improved. He continued to have a mild right INO. The right facial weakness had resolved. Dysarthria was improved and only mild. His left‐sided hemiparesis was mild and improved, but he had bilateral ataxia, worse on the left and was primarily using a wheelchair. Given his clinical and radiographic improvement, he completed a 1‐year course of rifampin, isoniazid, and levofloxacin. Surgical repair of his TOF was on hold pending completion of TB therapy and continued risk–benefit assessment based on the extent of his neurologic recovery.

## 3. Discussion

TOF is a relatively common congenital heart anomaly accounting for 3.5% of congenital heart defects (CHDs) [1] and was first described by Etienne Fallot in 1888. The combination of cardiac defects usually leads to pulmonary hypertension and cyanosis with a high risk for subsequent cardiac and other organ pathology. Since the development of the palliative Blalock–Taussig shunt in 1944 and definitive surgical correction in 1954, most individuals with TOF have undergone surgery in infancy. No large studies of life expectancy with uncorrected TOF have been published since a 1978 review [2] of 216 Danish autopsy findings up to 1949, alongside an additional 566 cases identified from the literature. The authors concluded that only 24% of persons with uncorrected TOF survive to age 10 years, with a continued decline in survival such that only 3% reach age 40. Advances in the medical treatment of heart disease since then may have improved these outcomes. However, more recent series of cases from resource‐poor regions reporting uncorrected TOF in adults [7–9] typically involve surgical correction after diagnosis, and no data are available for mortality rates before surgery or follow‐up of individuals unable to have a corrective procedure.

Individuals with TOF are prone to a number of infectious complications both before and after surgical correction. A large study of 1164 Danish patients with TOF found an incidence of infective endocarditis of 22.4–46.7 per 10,000 patient years compared to 0.1 per 10,000 patient years in controls [4]; however, most of these patients had undergone surgical repair, frequently including pulmonic valve replacement. Among patients who undergo late repair, a 2012 Taiwanese study of 179 patients who had first repair between ages 10 and 49 years revealed the following diagnoses before surgery: infective endocarditis (6.7%), brain abscess (4.6%), and pulmonary TB (3.3%) [8]. There are rare reports of patients in their sixth or seventh decade presenting with uncorrected TOF [10]. One such report described a 56‐year‐old woman presenting with shunt infective endocarditis and pulmonary embolism [11]. Our patient, at 63 years old, was similarly uncorrected yet remained largely asymptomatic from a cardiopulmonary standpoint.

Patients with cyanotic congenital heart disease, including TOF, are at higher risk of intracranial abscesses [6]. A German study [5], which followed 483 such patients prospectively, found that brain abscesses were common, with an incidence of 0.45%/year, with TOF patients carrying the highest cumulative risk of 12.1 ± 1.7% within the first 2 decades of life. Brain abscesses in cyanotic congenital heart disease are primarily attributed to right‐to‐left shunting [6], which allows hematogenous spread of bacteria directly into the systemic circulation, bypassing the pulmonary phagocytic filter. Furthermore, there is a dose–response relationship between shunt magnitude and brain abscess, with larger shunts carrying increased risk of cerebral abscess [12]. In addition, compensatory polycythemia from chronic hypoxemia increases blood viscosity and promotes cerebral microvascular stasis; it has been proposed that the resulting microinfarcts may disrupt the blood–brain barrier and create a nidus for CNS infection [13]. Tuberculous vasculitis also has been proposed in other cases of stroke in the setting of TB [14].

The differential diagnosis of a brain abscess in a patient with TOF is broad and must be approached systematically. Pyogenic bacteria are the most common causes of endocarditis and brain abscess in individuals with TOF. Endocarditis is usually readily diagnosed by blood cultures, and bacterial brain abscesses should respond to antibacterial therapy that crosses the blood–brain barrier. Blood cultures were negative in our patient’s case. Fungal organisms can also cause brain abscesses, notably *Aspergillus* species, but would be uncommon in the absence of significant immunosuppression or the rare case of fungal endocarditis. Parasites, including the tapeworm *Taenia solium,* which causes cysticercosis, and ameba can cause brain abscesses, but are extremely rare causes and may have specific diagnostic clues on CNS imaging. Finally, primary or metastatic malignancy involving the CNS can undergo central necrosis and appear more like an abscess than a solid mass; lung carcinoma could cause the radiographic findings seen in our patient’s head and chest studies and was an early diagnostic consideration.

While TB is an uncommon diagnosis in the United States, it is one of the most common pulmonary diseases in developing countries. Of the cases of TB reported in the United States in 2022, 73.8% were foreign‐born, and 10% had extrapulmonary as well as pulmonary TB [15]. Notably, in one study of extrapulmonary TB involvement, nearly half of the chest radiographs did not show abnormalities [16]. The hematogenous spread of *Mycobacterium TB* (*M. tb*) can lead to enlarging CNS granulomatous lesions named tuberculomas. The mass‐like lesions may undergo necrosis, resulting in abscesses, which usually have a contrast‐enhancing thin rim on CT scanning [17]. The lesions often have marked perilesional edema, but their appearance may differ based on the maturation stage [18, 19]. CNS tuberculomas are relatively rare, and a brainstem location is the least common for intracranial tuberculomas. Large CNS tubercular lesions over 3 cm in diameter and abscesses are relatively resistant to antituberculous therapy [17]. Our patient’s CNS TB lesion was 2.9 cm in greatest diameter with septation and ring‐enhancement and has been classified as a tuberculous abscess in this manuscript.

A search of the literature found seven cases of a TB CNS lesion associated with TOF, all uncorrected surgically. The characteristics of these individuals are summarized in Table 1. Five were children aged 11 years or younger [20–24], one was a 15‐year‐old boy [25], and one was a 23‐year‐old adult man [26]. Of the six cases in which CNS TB location was reported, five were in the cerebrum, and only the 23‐year old had a brainstem location (pons). Five of the individuals had culture, serologic, or histopathologic evidence of TB to guide treatment; two received empiric antituberculous therapy. Outcomes were as follows: four improved (though sometimes with residual neurologic deficits), one died, and two were lost to follow‐up, or the outcome was not reported.

**TABLE 1:** Case reports of individuals with TOF and TB CNS lesions.

Patient	TOF status	CNS lesion/confirmation	Associated diagnoses	Outcome/follow‐up	Ref/date
2 y.o. male, from India	Uncorrected (status uncertain)	Two frontoparietal abscesses, 3 × 3.5 cm; abscess material AFB smear (−), culture (+) *M. tuberculosis*	Not reported	Lost to follow‐up	Abraham et al.[20], 2009
7 y.o. male, from Philippines	Uncorrected (status uncertain)	TB meningitis with tuberculoma; site not specified; CSF culture (+) *M. tuberculosis*	Not known	Not known	Desphy et al. [21], 2000
9 y.o. female, from India	Uncorrected; managed medically since infancy	Mixed attenuation frontoparietal lesion; dry aspirate; postmortem pathology (+) granulomatous lesion consistent with TB; positive Mantoux skin test	Cyanosis since infancy; hypertrophic osteoarthropathy of elbows and knees	Died in the hospital on Day 25	Ray et al. [22], 1988
10 y.o. male, from Iran	Uncorrected (status uncertain)	Multifocal abscesses; CSF PCR and culture (+) *M. tuberculosis*	Cardiac medications discontinued prior to admission for economic reasons	Survived with neurologic complications	Khodabandeh et al. [23], 2017
11 y.o. male, from Nepal	Uncorrected; TOF diagnosed at age 5	Multiple bilateral cerebral ring‐enhancing lesions; improved within 10 days on anti‐TB therapy; admission blood cultures (+) *S. aureus*	Not reported	Neurologic deficit‐free at 2 months	Chamlagain et al. [24], 2021
15 y.o. male, from developing country	Uncorrected	Multiple large left parieto‐occipital abscesses; drainage specimen (+) TB PCR	Asplenia	Significant abscess reduction on MRI at 3 months	Desai et al. [25], 2013
23 y.o. male, from India	Uncorrected; diagnosed 1 year prior to admission	1.8 × 1.6 × 1.2 cm ring‐enhancing pontine lesion; biopsy (+) necrotic tissue with chronic inflammation	Not reported	Pontine lesion nearly resolved on CT at 6 months; no significant neurologic impairment at 26 months	Moorthy et al. [26], 2003

*Note:* TB = tuberculosis.

Abbreviations: AFB = acid‐fast bacilli; CNS = central nervous system; CT = computerized tomography; MRI = magnetic resonance imaging; PCR = polymerase chain reaction; TOF = tetralogy of Fallot.

Congenital heart disease has been associated with the development of TB, though this depends on the flow characteristics of the lesion and the degree of cyanosis present [27]. This may be due to a generally weakened health status as well as the greater likelihood that individuals with uncorrected heart defects in resource‐poor regions of the world are exposed to TB. The diagnosis of TB in the CNS is challenging, whether tuberculoma, tuberculous abscess, tuberculous radiculomyelitis, or TB meningitis. Due to TB’s predilection for the skull base, an invasive diagnostic procedure is not feasible in many cases. An aspirate or biopsy of our patient’s lesion would have allowed obtaining cultures and pathology. This could have yielded a definitive diagnosis and excluded other processes. However, the location of the suspected abscess in the brainstem greatly increased the risk of procedure‐related complications due to direct trauma, bleeding, and subsequent edema. A study of 1784 stereotactic CNS biopsies from 2009 to 2020 included 50 consecutive brainstem biopsies [28]. While diagnostic yield was high for brainstem biopsies (86%), symptomatic complications were higher at 20% vs. 0%, and mortality was higher at 6% vs. 0% for this location compared to cerebrum/cerebellum biopsies. Definitive confirmation of TB is also difficult, as AFB smear sensitivity for CNS TB is poor, and CSF mycobacterial cultures vary in yield. Clinical algorithms have been developed for CNS TB [29], but these have not been validated through prospective trials. Therefore, treatment decisions are often based on patient risk factors and clinical suspicion. While our patient did not undergo a biopsy of the pontine lesion, he did have a positive QuantiFERON assay despite negative AFB smears and cultures on CSF and BAL. Therefore, the decision to initiate antituberculosis therapy was made, given clinical suspicion for CNS TB and confirmed by subsequent improvement in the patient’s clinical status.

The acute right cerebellar lesion evolved on MRI as would be expected for an uninfected infarct and appeared chronic on follow‐up imaging. There is no way to be certain of its etiology. However, it could have resulted from an inflammatory state with hypercoagulability from CNS tuberculous infection [30], vasculitis associated with TB [14], or bland cardioembolism from the unrepaired TOF flow disruption with right‐to‐left shunt. There was no radiographic evidence of large vessel stenosis proximal to the infarct, nor were there culture findings suggestive of infective endocarditis and a septic embolus.

Treatment of tuberculomas and CNS TB in general involves antituberculous therapy and adjunctive corticosteroids. Steroids are generally discontinued when the risk of surrounding edema has passed. For tuberculomas, standard treatment duration can range from 9 to 12 months, and resolution of the tuberculoma depends on the initial size, with 50% of lesions greater than 2.5 cm taking over 12 months to resolve [31]. Selecting a regimen to treat a suspected brain abscess and possible tuberculous CNS lesion has become more complicated. Lack of response to antibiotics effective against pyogenic bacteria has been taken as evidence of a nonbacterial etiology. Similarly, improvement on antituberculous therapy has been felt to strongly support a tuberculous etiology, as in our patient’s case. However, a nonspecific effect of high‐dose corticosteroids must also be considered if these are part of the regimen. The development and spread of highly drug‐resistant *M. tb* strains also complicates assessment of responses to empiric therapy. The fluoroquinolone levofloxacin and the oxazolidinone linezolid were used as part of initial antituberculous therapy in our patient because of concern over possible multidrug‐resistant *M. tb.* These drugs also have broad antibacterial and gram‐positive antibacterial activity, respectively. Both were continued longer term in combination with the patient’s more standard RIPE antituberculous therapy because of the chance that they had contributed to the response of the patient’s CNS abscess to treatment. Thus, a bacterial brain abscess could respond to some newer antituberculous drug regimens, while certain fluoroquinolones would be the mainstay of therapy if multidrug‐resistant *M. tb* was strongly suspected or confirmed as the pathogen [17, 32].

## 4. Conclusion

Complex cases involving rare diagnoses should be approached systematically, evaluating potential diagnoses in order of treatment urgency and utilizing invasive procedures based on patient risk–benefit. This case highlights the importance of awareness of the potential complications of untreated TOF as well as suspicion of TB in the proper clinical setting. CNS TB should be considered if there are signs and symptoms consistent with neurologic involvement or if there are suggestive findings of TB elsewhere. This is especially important if the patient is from a TB endemic area or is at high risk for TB in regions of lower TB burden. Anti‐TB therapy should not be delayed awaiting definitive diagnosis, as spinal fluid TB smear sensitivity is low, and cultures may take weeks to grow. Prompt treatment may prevent the substantial morbidity and risk of death from untreated CNS TB.

## Funding

The authors disclose receipt of financial support up to US$5000 for publication costs from the Sharpe‐Strumia Research Foundation (SSRF) of the Bryn Mawr Hospital. H.H.L. received a SSRF stipend included within this amount to provide mentorship for residents in training in the preparation of the manuscript.

## Disclosure

This case was presented at a hospital Morbidity and Mortality Conference, but has not been submitted elsewhere or previously published.

## Consent

Written informed consent was obtained for the individual whose case is being reported.

## Conflicts of Interest

The authors declare no conflicts of interest.

## Data Availability

Data sharing is not applicable to this article as no datasets were generated or analyzed during the current study.
